# Miltirone exhibits antileukemic activity by ROS-mediated endoplasmic reticulum stress and mitochondrial dysfunction pathways

**DOI:** 10.1038/srep20585

**Published:** 2016-02-05

**Authors:** Ling Zhou, Lifeng Jiang, Maolei Xu, Qun Liu, Ning Gao, Ping Li, E-Hu Liu

**Affiliations:** 1 State Key Laboratory of Natural Medicines (China Pharmaceutical University), No. 24 Tongjia Lane, Nanjing 210009, China; 2 School of Pharmacy, Binzhou Medical University, Guanhai Road 346, Yantai, Shandong 264003, China; 3 Department of Pharmacognosy, College of Pharmacy, 3rd Military Medical University, Chongqing 400038, China

## Abstract

In this study, we investigated the effects of miltirone in human leukemia cell lines, primary leukemia cells, and nude mice U937 xenograft. Treatment of cells with miltirone resulted in apoptosis, mitochondria membrane potential (MMP) collapses, increase of Bax/Bcl-2 ratio, and cytochrome c release. Miltirone triggered the endoplasmic reticulum (ER) stress identified through several key molecules of the unfolded protein response, including phosphorylated PERK, eIF2a, GRP78, GRP94, and caspase-12. Miltrone treatment also resulted in the release of Ca^2+^ from the ER stores and mitochondrial Ca^2+^ loading in the cells. Further research revealed that miltirone resulted in dose-dependent decrease in complex III activity and elevated reactive oxygen species (ROS) production in these cells. Miltirone-induced apoptosis, dissipation of MMP and ER stress were dramatically blocked by pretreatment with antioxidant N-acetylcysteine (NAC). In contrast, treatment with ER stress inhibitor TUDCA significantly attenuated miltirone-induced ROS and apoptosis in leukemia cells. Moreover, our *in vivo* findings showed that administration of miltirone markedly inhibited tumor growth and induced apoptosis in U937 xenograft model with low systemic toxicity. Taken together, these findings indicate that miltirone may exert its antileukemic activity by inducing apoptosis through a ROS-dependent destructive cycle involving ER stress and mitochondrial dysfunction.

Reactive oxygen species (ROS) profoundly impact a number of cellular responses, including protein kinase activation, cell cycle progression, and apoptotic cell death^1^,^2^. In eukaryotic cells, the mitochondrial electron-transport chain is the main source of ROS during normal metabolism. The rate of ROS production is increased under pathological conditions and chemical inhibition of mitochondrial respiration. Two sites in the electron-transport chain, complex I and complex III, have been suggested to be the major sites for ROS production^3^,^4^. Excessive or sustained ROS can cause damage to proteins and DNA via an array of nonenzymatic and enzymatic detoxification mechanisms, thereby disrupting their structure and altering their functions, and activate or inhibit related signaling pathways^5^. Therefore, the perturbation of ROS homeostasis is considered as a new strategy for cancer treatment.

The endoplasmic reticulum (ER) is a specialized organelle for the synthesis and post-translational modification of proteins, which is highly sensitive to changes in intracellular homeostasis and extracellular stimuli. In addition, the ER plays an important role in homeostasis of intracellular Ca^2+^ and redox balance. The ER and mitochondria build a dynamic network where they cooperate in the generation of Ca^2+^ signals^6^. Studies suggest that disturbances of ER Ca^2+^ homeostasis or protein processing can lead to ER stress, which could in turn induce the production of ROS in the ER and mitochondria^7^. High ROS generation within mitochondria also initiates a sequence of events involving a number of proteins regulating apoptosis by opening of the mitochondrial permeability transition pore (mPTP)^8^. The mPTP contributes to the initiation of cell death pathways, either by causing ATP depletion and energetic collapse or by promoting the release of cytochrome c and / or apoptosis-inducing factor (AIF)^9^.

Miltirone is a naturally occurring diterpene quinine compound isolated from Salvia miltiorrhiza (Fig. 1a), which has been reported to possess a wide pharmacological activities, including prevention of angina pectoris, myocardial infarction and anticancer^10^,^11^,^12^. Evidences support that miltirone exerts antiproliferative, antiplasmodial, antitrypanosomal, and antioxidant activities^13^,^14^. A recent study has shown that miltirone induces cell cycle arrest and apoptosis in CCRF-CEM acute lymphoblastic leukemia cells^15^. However, the molecular mechanisms of miltirone-induced apoptosis in human leukemia cells are not fully defined. Also, there is no available information concerning miltirone’s *in vivo* efficacy against leukemia.

In the present study, we report that miltirone exhibited antileukemic effect *in vitro* and *in vivo*. The role of ROS in miltirone-induced apoptosis was fully investigated. In addition, we provided evidence that miltirone induced ROS production by inhibition of mitochondrial respiratory chain complex III. The mitochondrial ROS production disturbed ER protein folding inducing ER stress and ER Ca^2+^ release, which further increases ROS production and mitochondrial dysfunction. These findings provide the detailed mechanistic basis for the application of miltirone in the treatment of leukemia.

## Results

### Miltirone induces cytotoxicity and apoptosis in human leukemia cells

**Figure 1:**
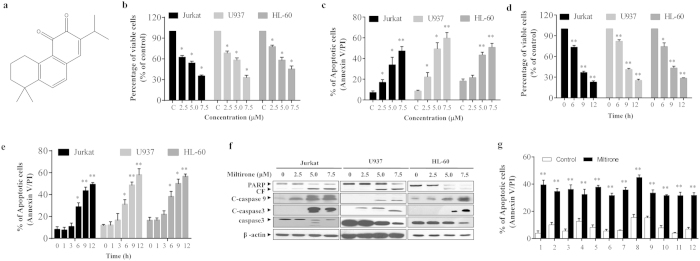
Miltirone markedly induces cytotoxicity and apoptosis in human leukemia cells in a dose- and time-dependent manner. (**a**) The chemical structure of miltirone. (**b**–**c**) Jurkat, U937 and HL-60 cells were treated with miltirone (0–7.5 μM) for 12 h, cell viability was analyzed by CCK8 assay (**b**) and the percentage of apoptotic cells was determined using flow cytometry (**c**). (**d**–**e**) Cells were treated with 7.5 μM miltirone for indicated time, cell viability was analyzed (**d**) and the percentage of apoptotic cells was determined (**e**). (**f**) Cells were treated with miltirone (0–7.5 μM) for 12 h, total cellular extracts were prepared and subjected to western blot analysis using antibodies against PARP, cleaved-caspase (C-caspase)-9, cleaved-caspase-3, caspase-3 and β-actin; n = 3. The bands were excised from different gels which were run under the same electrophoresis condition. (**g**) Primary leukemia blasts were isolated from six patients with AML (1–6) and six patients with ALL (7–12) as described in the Materials and Methods section. After exposure to 7.5 μM miltirone for 12 h, the extent of apoptosis was determined using flow cytometry. Data were presented as Mean ± SD. The differences were significant at *p < 0.05, **p < 0.01 *vs*. control. CF, cleaved fragment.

Dose response and time course analysis of miltirone-mediated proliferation inhibition and apoptosis in human leukemia cells (Jurkat, U937 and HL-60) are shown in Fig. 1. Moderate decrease in cell viability and increase in apoptosis were noted at 2.5 μM miltirone concentrations (12 h). The events became apparent at 5.0 μM and very extensive at 7.5 μM concentrations (Fig. 1b,c). Time-course analysis of cells exposed to 7.5 μM miltirone demonstrated a significant decrease in cell viability and increase apoptosis as early as 6 h. These events became apparent after 9 h of drug exposure, and reached near maximal levels after 12 h (Fig. 1d,e). Consistent with these findings, the same miltirone concentrations (2–7.5 μM) and exposure time (12 h) resulted in cleavage/activation of caspase-3/-9, and degradation of poly-ADP-ribose polymerase (PARP) (Fig. 1f).

To determine whether the effects of miltirone on apoptosis were restricted to human leukaemia cell lines, parallel experiments were carried out in primary leukemia blasts from six acute myeloid leukemia (AML) and six acute lymphoblastic leukemia (ALL) patients. These AML and ALL blasts were treated without or with 7.5 μM miltirone for 12 h, after which apoptosis were determined by Annexin V/PI analysis. As shown in Fig. 1g, treatment of AML and ALL cells with miltirone resulted in marked increase in apoptosis. These findings indicate that miltirone induces apoptosis in both human leukemia cell lines and primary leukemia blasts.

### Miltirone exhibits anticancer activity and low toxicity in U937 xenograft model

**Figure 2:**
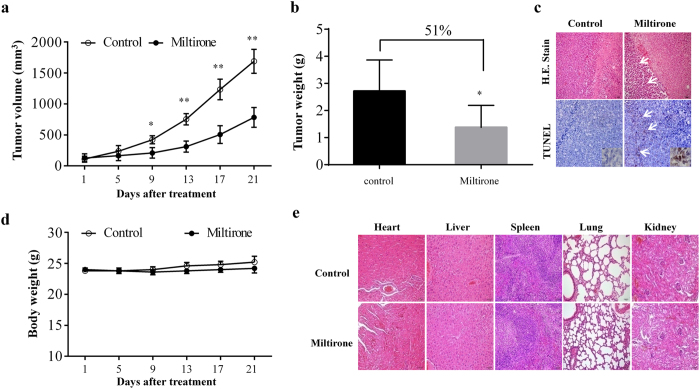
Miltirone has a potential of antitumor effect and low toxicity *in vivo*. The transplanted mice U937 leukemias were treated with 15 mg/kg of miltirone by i.p. every other day. Control group was treated with vehicle. (**a**) Tumor volume was measured every four days. (**b**) Tumor mass were weighed. (**c**) Tumors were fixed and stained with hemtoxylin and eosin (H&E) stain to examine tumor cell morphology, using TUNEL assay to determine apoptosis. (**d**) Body weight of mice during the 20 days of miltirone treatment. (**e**) H&E stained main organs of mice from treated and control sets to evaluate the toxicity of miltirone. Data were presented as Mean ± SD, n = 6. The differences were significant at *p < 0.05, **p < 0.01 *vs*. control.

The ability of miltirone to kill leukaemia cells *in vitro* led us to further evaluate its *in vivo* antitumor activity using U937 xenograft model. The tumor volume measurement further confirmed the significant reduction in the miltirone treatment group. As shown in Fig. 2a, treatment with miltirone significantly inhibited tumor growth 9 days following drug exposure (*p *= 0.0393 versus vehicle control). These events became more apparent 13, 17 and 21 days after drug exposure (*p *= 0.0088, 0.0086 and 0.0070 respectively, between miltirone treatment and vehicle control). There was a highly significant reduction (about 51%, *p *= 0.0429) in the mean resected tumor weight of the miltirone-treated group compared with vehicle-control mice (Fig. 2b).

We further determined the morphological changes and induction of apoptosis in tumor tissue of leukemia xenograft using H&E staining and TUNEL assay. As shown in Fig. 2c, the sections of U937 xenografts from mice treated with miltirone showed that cancer cells were markedly decreased, with signs of necrosis with infiltration of inflammatory cells. Furthermore, TUNEL-positive apoptotic cells of tumor sections from miltirone-treated mice significantly increased compared with the control group.

Meanwhile, there was no significant difference in the average body weight between the miltirone treatment and vehicle-control group (Fig. 2d). In addition, no morphological changes were observed in the organs of the tumor bearing mice that were treated with miltirone under H&E staining (Fig. 2e), indicating that no severe toxicity was observed.

### Miltirone triggers ROS generation

**Figure 3:**
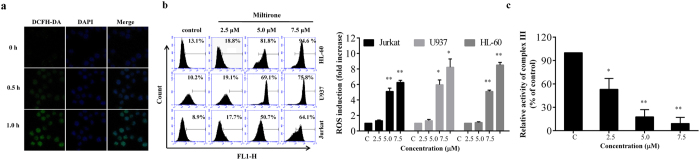
Miltirone induces the generation of ROS. (**a**) Jurkat cells were incubated with 7.5 μM miltirone for 0, 0.5 and 1 h and confocal images of intracellular ROS levels (green fluorescence) and cell nuclei stained with DAPI (blue fluorescence) were acquired using confocal microscopy. (**b**) Jurkat, U937, and HL-60 cells were incubated with miltirone (0–7.5 μM) for 1 h, and the level of intracellular ROS was measured by flow cytometry. (**c**) Jurkat cells were incubated with 7.5 μM miltirone for 1 h, mitochondrial fractions were isolated and complex III activity was monitored. Data were presented as Mean ± SD. The differences were significant at *p < 0.05, **p < 0.01 *vs*. control.

Increasing evidences suggest that ROS plays an important role in a variety of cell death mechanisms induced by widely used chemotherapeutics^16^. Next, we tested whether intracellular ROS is associated with miltirone-induced apoptosis in human leukemia cells. In Jurkat cells, the intracellular ROS levels were increased at 0.5 h and significantly increased as early as 1 h following exposure to 7.5 μM miltirone (Fig. 3a). To determine whether miltirone-induced ROS observed in Jurkat cells also occur in other leukemia cell lines, parallel studies were carried out. Exposure of Jurkat, U937 and HL-60 cells to miltirone (2.5–7.5 μM) for 1 h resulted in a pronounced increase of ROS (Fig. 3b). These data support our hypothesis that miltirone treatment leads to an increase in ROS production, which may represent a critical step in miltirone induced apoptosis in human leukemia cells.

To elucidate the source of ROS, the activities of complex I and complex III were detected as described in Materials and Methods. The data indicate that miltirone inhibited complex III activity in a dose dependent manner (Fig. 3c) while failed to decrease substantially complex I activity (Supplementary Fig. S1).

### ROS plays an important role in miltirone-induced apoptosis

**Figure 4:**
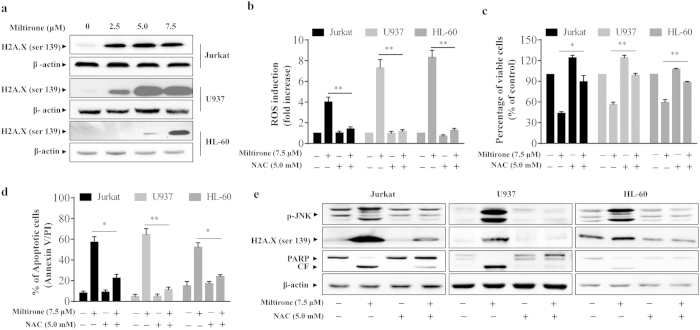
Role of ROS generation in miltirone induced apoptosis. (**a**) Jurkat, U937 and HL-60 cells were incubated with miltirone (0–7.5 μM) for 12 h, and the expression of H2A.X (ser 139) and β-actin was analyzed by western blotting; n = 3. The bands were excised from different gels which were run under the same electrophoresis condition. (**b**) Cells were treated with 7.5 μM miltirone for 1 h in the presence or absence of 5 mM NAC and the level of intracellular ROS was measured by flow cytometry. (**c**–**e**) Cells were treated with 7.5 μM miltirone for 12 h in the presence or absence of 5 mM NAC, cell viability (**c**), apoptosis (**d**) and the expression of p-JNK, H2A.X (ser 139), PARP, cleaved fragment (CF) of PARP and β-actin (**e**) were analyzed; n = 3. The bands were excised from different gels which were run under the same electrophoresis condition. Data were presented as Mean ± SD. The differences were significant at *p < 0.05, **p < 0.01 miltirone *vs*. co-treatment with miltirone and NAC.

Several recent studies have shown that generation of ROS by diverse cell death stimuli does not only initiate cascades of cell death signals but also directly lead to DNA damage^17^. To assess the effect of miltirone on DNA damage, the alkaline comet assay was employed in Jurkat cells. Compared with controls, an obvious characteristic “comet” migration pattern of relaxed DNA was observed in miltirone-treated cells (Supplementary Fig. S2). Overall, comet assay results clearly showed DNA-damaged Jurkat cells following miltirone treatment. Moreover, the phosphorylation of histone H2A.X at Ser 139, a marker for DNA damage, was detected by western blotting in three human leukemia cell lines. The results indicate that miltirone dramatically increased the phosphorylation of histone H2A.X in a dose-dependent manner (Fig. 4a). Following DNA damage, cells display complex dynamic phenotypes that connect cell-cycle arrest in G1, S, or G2/M phase, and DNA repair with decisions related to survival, cell-cycle reentry, permanent cell-cycle arrest, or cell death^18^. Next we assessed the effect of miltirone on cell-cycle progression in human leukemia cells. It was shown that miltirone (2.5–7.5 μM, treated for 12 h) arrested Jurkat and HL-60 cells in G2/M-phase, while U937 cells were arrested in G0/G1-phase (Supplementary Fig. S3). These findings suggest that the inhibition of cell growth by miltirone is associated with the DNA damage and induction of cell cycle arrest.

ROS have been demonstrated as an inducer or mediator for the activation of MAPK pathways that are responsible for ROS-mediated cell apoptosis^19^. To investigate the possible role of MAPK pathways in miltirone induced apoptosis, the expression of the phosphorylated forms of extracellular signal-regulated kinases1/2 (ERK1/2), p38, and c-Jun N-terminal kinase (JNK) was evaluated by western blot analysis. As shown in Supplementary Fig. S4a, miltirone induced a robust and sustained activation of JNK, but not p38 and ERK1/2, suggesting that JNK may be specifically activated in miltirone-induced apoptotic pathway. However, pretreatment with JNK inhibitor SP600125 failed to block the decrease cell viability of Jurkat and U937 cells mediated by miltirone (Supplementary Fig. S4b).

To further confirm the functional role of ROS in miltirone-mediated lethality in leukemia cells, the ROS scavenger NAC was employed. Pretreatment with NAC almost completely inhibited the ROS generation induced by miltirone treatment (Fig. 4b). Significantly, NAC attenuated miltirone**-**mediated cytotoxicity and apoptosis (Fig. 4c,d). Furthermore, NAC pretreatment could effectively block the ability of miltirone to promote the phosphorylation of H2A.X and JNK and the degradation of PARP (Fig. 4e). These data indicate that increase of ROS has a critical role in miltirone-induced apoptosis in leukemia cells.

### Miltirone induces the ER stress response of human leukemia cells

**Figure 5:**
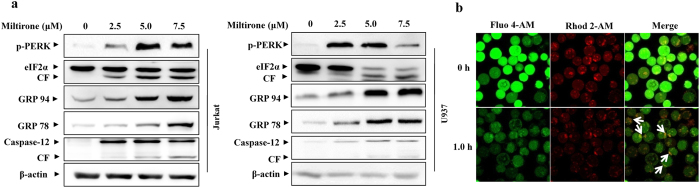
Miltirone induces ER stress in Jurkat and U937 cells. (**a**) Shown are the effects of miltirone on the expression of ER stress-related proteins. Jurkat and U937 cells treated with miltirone at indicated concentrations for 12 h, whole-cell lysates were obtained and subjected to western blot analysis using antibodies against p-PERK, eIF2α, GRP78, GRP94, caspase-12 and β-actin; n = 3. The bands were excised from different gels which were run under the same electrophoresis condition. (**b**) Jurkat cells were treated with miltirone for 1.0 h, change of ER-Ca^2+^ levels (green) was observed using confocal microscopy. White arrows in 1.0 h panel indicate residual ER-Ca^2+^ are redistributed and trafficked into mitochondria.

Studies suggest that altered redox homeostasis in the cell is sufficient to cause ER stress, which could in turn induces the production of ROS in the ER and mitochondria^20^. To test whether miltirone induces ER stress in human leukemia cells, we examined the ER stress markers expression after treatment with miltirone in Jurkat and U937 cells. As shown in Fig. 5a, miltirone (2.5–7.5 μM) effectively triggered the expressions of ER stress related molecules including phosphorylated PERK (p-PERK), eIF2a, GRP78, GRP94, and caspase-12 in these cells in a dose-dependent manner. These results indicate that miltirone is capable of inducing the ER stress in human leukemia cells.

A growing body of literature suggest that mitochondrial ROS production increases the ER Ca^2+^ release and mitochondrial Ca^2+^ loading^21^,^22^. We therefore asked whether miltirone treatment could result in changes in intracellular Ca^2+^ fluxes. The ER-Ca^2+^ can be detected using Fluo 4-AM (green channel), and mito Ca^2+^ was detected using Rhod 2-AM (red channel), a dye that preferentially accumulates in mitochondria. As shown in Fig. 5b, Fluo 4-AM clearly labelled the ER network in Jurkat cells at 0 h; after miltirone treatment for 1 h, the significantly decreased signal indicated that miltirone could promote the Ca^2+^ release from the ER stores. Of note, ER Ca^2+^ trafficking into mitochondria were observed following treatment with miltirone for 1 h and this phenomenon was not observed in the control group (Supplementary Fig. S5).

### Miltirone triggers apoptosis through the mitochondrial dysfunction pathway

**Figure 6:**
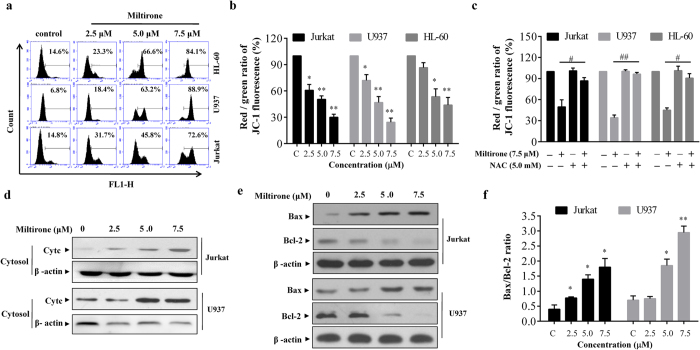
Miltirone triggers the mitochondrial dysfunction pathway. (**a**–**b**) Exposure of Jurkat, U937, and HL-60 cells to miltirone (0–7.5 μM) for 3 h caused disruption of MMP as evidenced by an increase in the proportion of cells with green fluorescent light (**a**) and decrease in the proportion of cells with higher red (JC-1 aggregates) / green (JC-1 monomers) ratio of JC-1 fluorescence (**b**). (**c**) Cells were treated with 7.5 μM miltirone for 3 h in the presence or absence of 5 mM NAC and the dissipation of MMP was measured. (**d**–**e**) Jurkat and U937 cells were treated with miltirone (0–7.5 μM) for 12 h, cytosolic fractions (**d**) and whole-cell lysates (**e**) were obtained and subjected to western blot analysis using antibodies against cytochrome c (Cyt c), Bax, Bcl-2 and β-actin; n = 3. The bands were excised from different gels which were run under the same electrophoresis condition. (**f**) A remarkable Bax / Bcl-2 ratio increasing was demonstrated. Data were presented as Mean ± SD. The differences were significant at *p < 0.05, **p < 0.01 *vs*. control and # *p *< 0.05, ## *p *< 0.01 miltirone *vs*. co-treatment with miltirone and NAC.

Increased levels of mitochondrial Ca^2+^ stimulate mitochondrial ROS formation, which causes the mPTP to open and then the mitochondria membrane potential (MMP) collapses completely^22^,^23^. To investigate the effect of miltirone on mitochondrial function, we first measured MMP using the JC-1, a fluorescent dye that accumulates selectively within mitochondria depending on the membrane potential. Exposure of human leukemia cells to miltirone (2.5–7.5 μM) for 3 h caused disruption of MMP as evidenced by an increase in the proportion of cells with green fluorescent light and decrease in the proportion of cells with higher red/green ratio of JC-1 fluorescence (Fig. 6a,b). Pretreatment with NAC effectively inhibited the MMP disruption by miltirone (Fig. 6c).

Since cytochrome c release is linked to the loss of MMP, we next examined the distribution of cytochrome c in leukemia cells after miltirone exposure. Cytosolic and mitochondrial fractions were prepared from cells exposed to miltirone (2.5–7.5 μM) for 12 h and cytochrome c was detected by western blot analysis. As shown in Fig. 6d, treating cells with miltirone resulted in accumulated cytochrome c in the cytosol in a dose-dependent manner. The expression of Bax and Bcl-2 were also measured by western blot analysis. Fig. 6e showed a dose-dependent suppression of Bcl-2 expression, accompanied by concomitant increases in Bax in miltirone-treated cells, leading to a raise in the Bax/Bcl-2 ratio (Fig. 6f). These results indicat that miltirone could induce a mitochondrial-mediated apoptotic pathway in human leukemia cells.

### Crosstalk between ER and mitochondrial involves in the apoptosis induced by Miltirone

**Figure 7:**
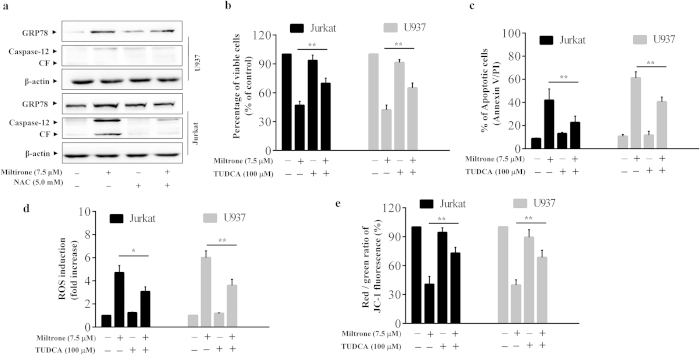
ROS-dependent ER stress is involved in the miltirone-mediated ROS production and apoptosis. (**a**) Cells were treated with 7.5 μM miltirone for 12 h in the presence or absence of 5 mM NAC and the expression of GRP78, caspase-12 and β-actin was analyzed; n = 3. The bands were excised from different gels which were run under the same electrophoresis condition. (**b**–**e**) Cells were treated with 7.5 μM miltirone for 12 h in the presence or absence of 100 μM TUDCA and the cell viability (**b**), apoptosis (**c**), ROS (**d**) and MMP (**e**) were detected. Data were presented as Mean ± SD. The differences were significant at *p< 0.05, **p< 0.01 miltirone *vs*. co-treatment with miltirone and TUDCA.

Evidence suggests that mitochondrial ROS generation could initiate a destructive cycle involving ER protein misfolding, Ca^2+^ release from ER stores and mitochondrial Ca^2+^ loading, which further increase ROS production^24^,^25^. To elucidate the mechanism of miltirone-induced ROS generation, we tested the effect of NAC on the changes of ER stress. Notably, pretreatment with 5 mM NAC for 1 h could effectively block miltirone-induced expression of GRP78 and caspase-12 in Jurkat and U937 cells (Fig. 7a). Such findings suggest that ER may be the target of ROS and ER stress is not the original cause of miltirone-induced ROS.

To investigate the possible role of ER stress in miltirone induced apoptosis, ER stress inhibitor, tauroursodeoxycholic acid (TUDCA) was used to alleviate the ER stress. Treatment of cells with TUDCA significantly attenuated miltirone-mediated cytotoxicity and apoptosis (Fig. 7b,c). Lastly, we investigated the effect of TUDCA on the ROS production and MMP collapse mediated by miltirone. Treatment with TUDCA effectively blocked miltirone-induced ROS and MMP loss (Fig. 7d,e). Such findings suggested a cross-talk of apoptotic signaling between the ER and mitochondria, which is involved in miltirone-induced apoptosis.

## Discussion

In this study, we provide that miltirone shows significant inhibitory effect on the growth of human leukemia cell lines and primary leukemia cells *in vitro* and the U937 xenografts *in vivo*. Our results also provide detailed mechanistic information as to how miltirone exerts its apoptotic effects on human leukemia cells (i.e., inhibition of complex III activity, ROS production, ER stress and mitochondrial dysfunction).

A number of studies implicate that ROS toxicity is an effective means of selectively eradicating malignant cells^26^. Miltirone, a less polar compound possessing an orthoquinone moiety in the molecule, shows antioxidant activity^27^. However, treatment of cells with miltirone could induce a pronounced increase of ROS, which is consistent with previous reports^15^,^16^,^17^,^18^,^19^,^20^,^21^,^22^,^23^,^24^,^25^,^26^,^27^,^28^. A critical question then arises regarding the mechanism by which ROS elevation occurs during miltirone treatment. Evidence suggests that mitochondria are important sites of ROS production in mammalian cell^29^. In order to elucidate the source of ROS, the activities of complex I and complex III, which were considered as the major sites for ROS production in mitochondria, were detected in this study. The results showed that miltirone treatment results in dose-dependent decrease in complex III activity and elevated ROS production. However, complex III is a large multienzyme complex composed of several subunits and the precise mechanism of this suppression by miltirone awaits further study.

Extensive evidence is accumulating that ROS can disturb ER protein folding and induce ER stress, which activates the unfolded protein response (UPR) to resolve this protein-folding defect^30^. In general, ER stress triggers three major branches of UPR including the PERK-eIF2, IRE1-XBP1 and ATF6 pathway, which serve as proximal sensors of protein folding status in the ER^31^. However, when the ER functions are impaired beyond restoration, pro-apoptotic signaling pathways are activated to protect the organism by eliminating damaged cells. Caspase-12 is an ER-resident caspase that is activated to mediate apoptosis^32^. Moreover, the presence of ROS also affects the Ca^2+^ homeostasis which is followed by the activation of the transcription of ER chaperone genes, such as GRP78/BiP and GRP94, to prevent cellular calcium toxicity^33^. In the present study, western blot analysis revealed that exposure of Jurkat and U937 cells to miltirone increased the expressions of p-PERK, GRP78, GRP94 and caspase-12. However, miltirone resulted in reduction of eIF2α and the production of its cleavage fraction. These findings are consistent with previous study, which demonstrated that cleavage of eIF2α could contribute to inhibition or alteration of protein synthesis during apoptosis in leukemia cells^34^. Moreover, miltirone stimulated ER Ca^2+^ release resulting in rapid increases in mitochondrial Ca^2+^. Excessive mitochondrial Ca^2+^ accumulation has been linked to further increase the rate of ROS production, mitochondrial dysfunction and precipitating apoptotic cell death^35^. In order to elucidate the involvement of ER stress in ROS generation, mitochondrial dysfunction and apoptosis mediated by miltirone, we ameliorated ER stress by treatment of TUDCA, a chemical chaperone which is known to inhibit the UPR. Our data indicated that treatment with TUDCA significantly attenuated miltirone-induced ROS, dissipation of MMP and apoptosis in leukemia cells. Evidence suggests that oxidative protein folding is an important resource of ROS production in the cell^36^. However, miltirone-activated UPR is suppressed by the antioxidant NAC, suggesting miltirone-mediated ER stress induction is oxidative stress-dependent. These results argue against the possibility that ER is the direct source of ROS during miltirone induced ROS production. Such findings are accordant with the studies that mitochondrial ROS generation initiates a destructive cycle involving ER stress and mitochondrial Ca^2+^ loading, which further increases ROS production and culminates necrotic cell death^24^,^25^.

The mPTP can initiate pathways to cell death, either by causing energetic collapse or by promoting the release of cytochrome c and/or apoptosis-inducing factor (AIF)^9^. ROS are key inducers of mPTP opening, which ultimately progress to the collapse of MMP in the whole mitochondrial population^37^. MMP disruption has been implicated in a variety of apoptotic phenomena, such as cytochrome c release and caspase activation. The ratio of Bax/Bcl-2 usually represents the degree of mitochondrial outer membrane permeabilization^38^. In this study, the rapid loss of MMP, release of cytochrome c, an increased Bax/Bcl-2 expression ratio and caspase-3/-9 activation were observed in cell lines after miltirone exposure. However, the loss of MMP was effectively blocked by NAC, indicating that miltirone-induced apoptosis in human leukemia cells is mediated by a ROS-dependent mitochondrial pathway.

ROS may interact with cellular DNA, leading to DNA damage which triggers a specific DNA damage response (DDR), including activation of any sensor kinases and phosphorylation of adaptor protein 53BP1 and histone H2A.X^39,40^. Finally, DDR activation leads to cells display complex dynamic phenotypes that connect cell-cycle arrest in G1, S, or G2/M phase^17^. Our studies revealed that miltirone could result in DNA strand breaks in human leukemia cells, as evident by the production of comet tails (Supplementary Fig. S2) and phosphorylation of H2A.X at ser 139, resulting in irreversible arrest of leukemia cells either at the G1 to S-phase boundary or G2/M-phase (Supplementary Fig. S3). It has been reported that increased ROS production in leukemic cells leads to the activation of MAPKs and cell death^41^. Three major MAPKs have been identified, including JNK, p38 and ERK. Our study indicates that the ROS-mediated JNK activation plays a role in the sensitization of human leukemia cells to miltirone (Supplementary Fig. S4a). However, the JNK inhibitor failed to block the miltirone mediated decrease cell viability significantly (Supplementary Fig. S4b), suggesting that activation of JNK is not absolutely important involved in the miltirone-regulated apoptosis.

In summary, the present findings demonstrate that miltirone effectively induces apoptosis in human leukemia cell lines and primary leukemia cells *in vitro* and leukemia xenograft *in vivo*. This effect occurs in association with the production of ROS. Miltirone induces apoptosis in human leukemia cells through a ROS-dependent destructive cycle involving ER stress and mitochondrial dysfunction, which further increases ROS production. We speculate that miltirone-induced ROS by disturbing mitochondrial complex III activity are key inducers of this destructive cycle. These results suggest that miltirone may be a promising agent for the treatment of hematologic malignancies.

## Materials and Methods

### Chemicals and reagents

Miltirone was separated from the extract of Salvia miltiorrhizae Radix. N-acetyl cysteine (NAC) was from Beyotime (Haimen, China). TUDCA was purchased from Sigma (Sigma-Aldrich, St. Louis, MO). Antibodies against PARP, caspase-3, cleaved-caspase-3, cleaved-caspase-9, H2A.X (ser 139), cytochrome c, BAX, Bcl-2, p-PERK, eIF2α, caspase-12, GRP78, GRP94, p-JNK and β-actin were purchased from Cell Signaling Technology (Beverly, MA, USA).

### Cell culture

U937, HL-60 and Jurkat cells were purchased from American Type Culture Collection (ATCC, Manassas, VA) and maintained in RPMI 1640 medium containing 10% fetal bovine serum (FBS). Cells were cultured at 37 °C in a humidified atmosphere and 5% CO_2_ in air.

Peripheral blood samples were obtained from six patients with acute myeloid leukaemia (AML) and six patients with acute lymphoma leukaemia (ALL) after informed consent. Approval was obtained from the Southwest Hospital (Chongqing, China) institutional review board for these studies. AML and ALL blasts were isolated by density gradient centrifugation over Histopaque-1077 (Sigma Diagnostics, St. Louis, MO, USA) at 400×g for 38 min as previously described^42^. Isolated mononuclear cells were washed and assayed for total number and viability using Trypan blue exclusion. Blasts were suspended at 8 × 10^5 ^ml^−1^ and incubated in RPMI 1640 medium containing 10% FBS in 24-well plates.

### Measurement of cell viability

Cell proliferation of human leukemia cells was detected using Cell Counting Kit-8 (Donjindo Molecular Technologies, Inc., MD) according to the manufacturer’s instruction. Briefly, 2 × 10^4^ cells/well in a 96-well plate were incubated with or without various concentrations miltirone for 0, 6, 9 or 12 h, 10 μl of CCK-8 solution was added to each well, and cells were incubated at 37 °C for 1–4 h. The absorbance was measured at 450 nm using a Universal Microplate Reader (BIO-TEK instruments, Inc., Vermont, MA).

### Apoptosis assay

The cells were stained with Annexin V-FITC / propidium iodide (PI) (BD Pharmingen, San Diego, CA, USA) and measured by flow cytometer according to the manufacturer’s instructions. Both early apoptotic (Annexin V^+^/PI^−^) and late apoptotic (Annexin V^+^/PI^+^) cells were considered as apoptotic cells.

### Measurement of ROS

The production of intracellular ROS was detected using fluorescent dye 2, 7-dichlorofluoresce diacetate (DCFH-DA, Beyotime Institute of Biotechnology, China). Briefly, 5 × 10^6^ cells/well cultured in a 6-well plate were incubated with or without various concentrations miltirone for indicated time. The cells were then treated with 10 μM DCFH-DA 30 min in the dark and measured for the oxidation of DCFH-DA using confocal microscopy or flow cytometer. The fluorescent intensity measuring the oxidation of DCFH-DA by ROS represents the relative steady state of ROS generation in cells.

### Enzyme assays

Cytochrome c reductase (complex III) activities were measured using Enzyme Activity Assay Kit (GENMED SCIENTIFICSINC, USA). Mitochondrial fractions were isolated using mitochondria Isolation Kit (Beyotime, Haimen, China) according to the manufacturer’s instructions and complex III activity was monitored by measuring the conversion of oxidized cytochrome c into reduced cytochrome c (absorption peaks at 550 nm).

### Mitochondrial Membrane Potential (MMP) measurement

The disruption of MMP was measured using fluorochrome dye JC-1 by flow cytometry. After treatment, the cells were harvested and incubated with JC-1 in a cell incubator for 20 min. We calculated MMP as a ratio between red- and green positive cells for the indicated periods of time using the flow cytometry (FACS Calibur, Becton Dickinson), and analyzed by software Modfit and FlowJo 7 with settings of FL1 (green) at 530 nm and FL2 (red) at 585 nm.

### Ca^2+^ imaging

ER Ca^2+^ and mitochondrial Ca^2+^ (mito Ca^2+^) fluxes were monitored with confocal microscope using Fluo 4-AM and Rhod 2-AM respectively^43^. Jurkat cells were seeded into glass-bottom culture dishes at a density of 2 × 10^6^cells per dish. After routine drug treatment, cells were loaded with cell-permeant calcium indicators, 2 μM of Fluo 4-AM (DOJINDO Laboratories) and 2 μM of Rhod 2-AM (DOJINDO Laboratories), in HBSS for 30 min at 37 °C. The calcium distribution imaging was measured by confocal microscopy.

### Western blotting

Western analyses were performed as described previously^44^. Briefly, whole cell lysates were extracted using radioimmunoprecipitation assay (RIPA) buffer containing protease inhibitor cocktail and phosphatase inhibitor cocktail (Roche, Mannheim, Germany). The concentration of proteins was determined using enhanced BCA protein assay reagent (Beyotime, Haimen, China) and protein samples were separated by 10–12% SDS-PAGE. Protein was transferred to nitrocellulose and western blot analysis performed. Detection was performed by electro chemical luminescence (ECL).

### Xenograft model and immunohistochemical evaluation

Nude mice (5 weeks old) were supplied by Shanghai Laboratory Animal Limited Company. Animal experiments were approved by the Institutional Animal Care and Use Committee (IACUC) of China Pharmaceutical University (Nanjing, China). Mice (n =12) were injected with U937 cells (2 ×10^6^) with matrigel (100 μL) to the right flank of each mice. When average tumor volume reached 100 mm^3^, mice were randomly assigned in two groups of 6 mice per group: (a) vehicle group; (b) miltirone group (15 mg/kg, intraperitoneally every other day for 3 weeks). Tumor size and body weight were measured every four days. Tumor volumes were calculated according to the formula (width^2^ × length)/2. All animals were sacrificed immediately after 20 days of drug exposure.

### TUNEL assay

To assess apoptosis in tumor tissue sections, TUNEL labeling was performed using the *In Situ* Cell Death Detection kit (Beyotime, Haimen, China). Briefly, tumor tissue sections of formalin-fixed, paraffin-embedded specimens were dewaxed in xylene and rehydrated in a graded series of ethanol. The tumor samples were incubated with proteinase K (2 mg/ml), and the TUNEL staining was performed according to the manufacturer’s instructions.

### Ethical Standards

All participants were required to give a written, informed consent. The studies was approved by the ethical committee and conducted in accordance with the Helsinki Declaration and Good Clinical Practice guidelines of ICH.

### Statistical analysis

All data was represented as mean ± standard deviation (SD) for at least three independent experiments and representative examples are shown. Student’s t-test was used for statistical analysis. Statistical tests were carried out using PRISM (GraphPad Software, San Diego, CA, USA). *p *< 0.05 (*) or *p* < 0.01 (**) was considered significantly different.

## Additional Information

**How to cite this article**: Zhou, L. *et al.* Miltirone exhibits antileukemic activity by ROS-mediated endoplasmic reticulum stress and mitochondrial dysfunction pathways. *Sci. Rep.* **6**, 20585; doi: 10.1038/srep20585 (2016).

## Supplementary Material

Supplementary Information
