# Supplementary material for: Miltirone exhibits antileukemic activity by ROS-mediated endoplasmic reticulum stress and mitochondrial dysfunction pathways
Source: Sci Rep. 2016 Feb 5;6:20585. doi: 10.1038/srep20585 (PMC4742825; doi:10.1038/srep20585)
Supplement: Supplementary Information [file srep20585-s1.doc]

**Miltirone exhibits antileukemic activity by ROS-mediated endoplasmic reticulum stress and mitochondrial dysfunction pathways**

Ling Zhou ^a, b 1^, Lifeng Jiang ^a 1^, Maolei Xu ^a, b^, Qun Liu ^a^, Ning Gao ^c^, Ping Li ^a^ ^*^ and E-Hu Liu ^a^ ^*^

*^a^ State Key Laboratory of Natural Medicines (China Pharmaceutical University), No. 24 Tongjia Lane, Nanjing 210009, China*

*^b^ School of Pharmacy, Binzhou Medical University, Guanhai Road 346, Yantai, Shandong 264003, China*

*^c^. Department of Pharmacognosy, College of Pharmacy, 3rd Military Medical University, Chongqing 400038, China*

* To whom correspondence should be addressed. Tel:/Fax: +86-25-8327-1379

**Correspondence**: Dr. E-Hu Liu (liuehu2011@163.com) or Dr. Ping Li ([liping2004@126.com](mailto:liping2004@126.com))

^1^ The authors contributed equally to this work.

**Materials and Methods**

**Chemicals and reagents**

Miltirone was separated from the extract of Salvia miltiorrhizae Radix. JNK inhibitor SP600125 was from Beyotime (Haimen, China). Antibodies against p-JNK, p38, p-p38, ERK, p-ERK and β-actin were purchased from Cell Signaling Technology (Beverly, MA, USA).

**Methods**

**Enzyme assays**

NADH : ubiquinone oxidoreductase (complex I) activities were measured using Enzyme Activity Assay Kit (GENMED SCIENTIFICSINC，USA). Mitochondrial fractions were isolated using mitochondria Isolation Kit (Beyotime, Haimen, China) according to the manufacturer’s instructions. Complex I activity was monitored by measuring the conversion of NADH (absorption peaks at 340 nm) into NAD^+^.

**Alkaline Comet Assays**

DNA lesions in single cells were assessed using a comet assay kit (keyGEN BioTECH, Nanjing, China) as previously described[^1^](#_ENREF_35). In general, following desired treatments, cells were harvested and washed with PBS, 10 μL cell suspension containing 10^4^ cells were mixed with 75 μL of 0.7% molten low melting agarose at 37°C. The agarose-cell mixture was placed on a slide precoated with 0.6% normal agarose. After solidified at 4°C, the slide was lysed in prechilled lysis solution in the dark for 3 h. After denaturation in a freshly prepared electrophoresis buffer for 20 min, the slide was subsequently electrophoresed at 300 mA for 20 min. Then, it was immersed in neutralization buffer, dehydrated, air-dried and stained with PI for 10 min. The slide was visualized by a fluorescence microscope (Nikon, Japan).

**Cell cycle analysis**

Cell cycle analysis was performed on the harvested cell pellets fixed with 70% ethanol and stored at 4°C overnight. After washing, the cells were incubated with 50 μg/mL RNase A in 37°C water bath for 2 h, and stained with 50 μg/mL PI for 15 min. The cell cycle distribution was measured using the flow cytometer with Cell Quest and Modfit LT 3.0 software (Becton Dickinson, Franklin Lakes, NJ).


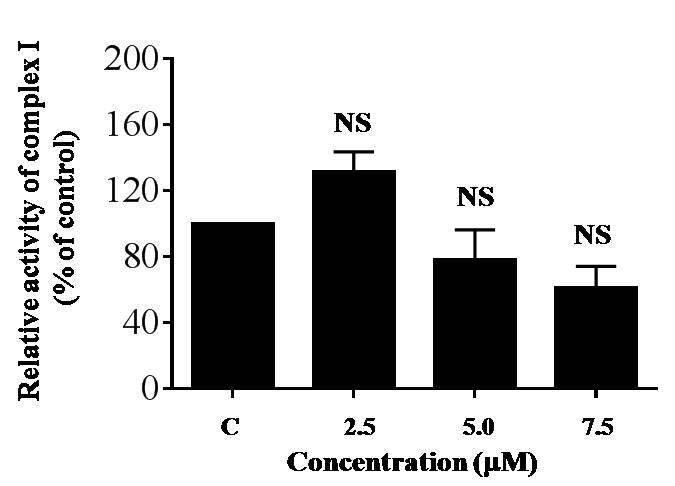


**Supplementary Fig. S1** **Activities of complex I were detected**. Jurkat cells were incubated with 7.5 μM miltirone for 1 h, mitochondrial fractions were isolated and complex I activity was monitored. Data were presented as Mean ± SD. The differences were no significant (NS) at *p* > 0.05 *vs*. control.


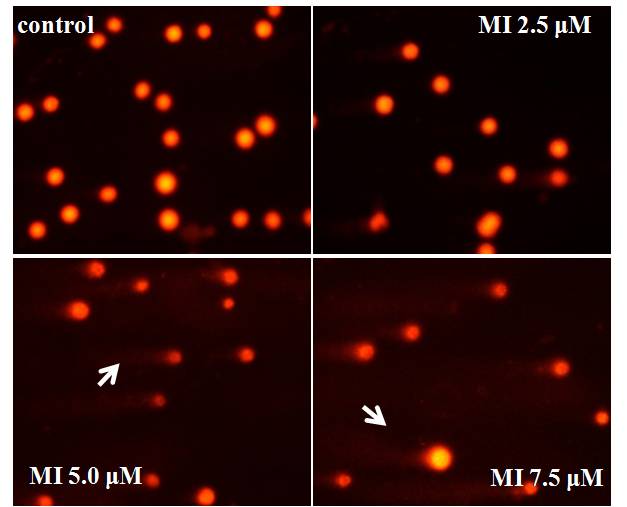


**Supplementary Fig. S2 Miltirone induces DNA damage in Jurkat cells.** Jurkat cells were incubated with miltirone (0-7.5 μM) for 6 h, and then comet assay was conducted as detailed in Methods.


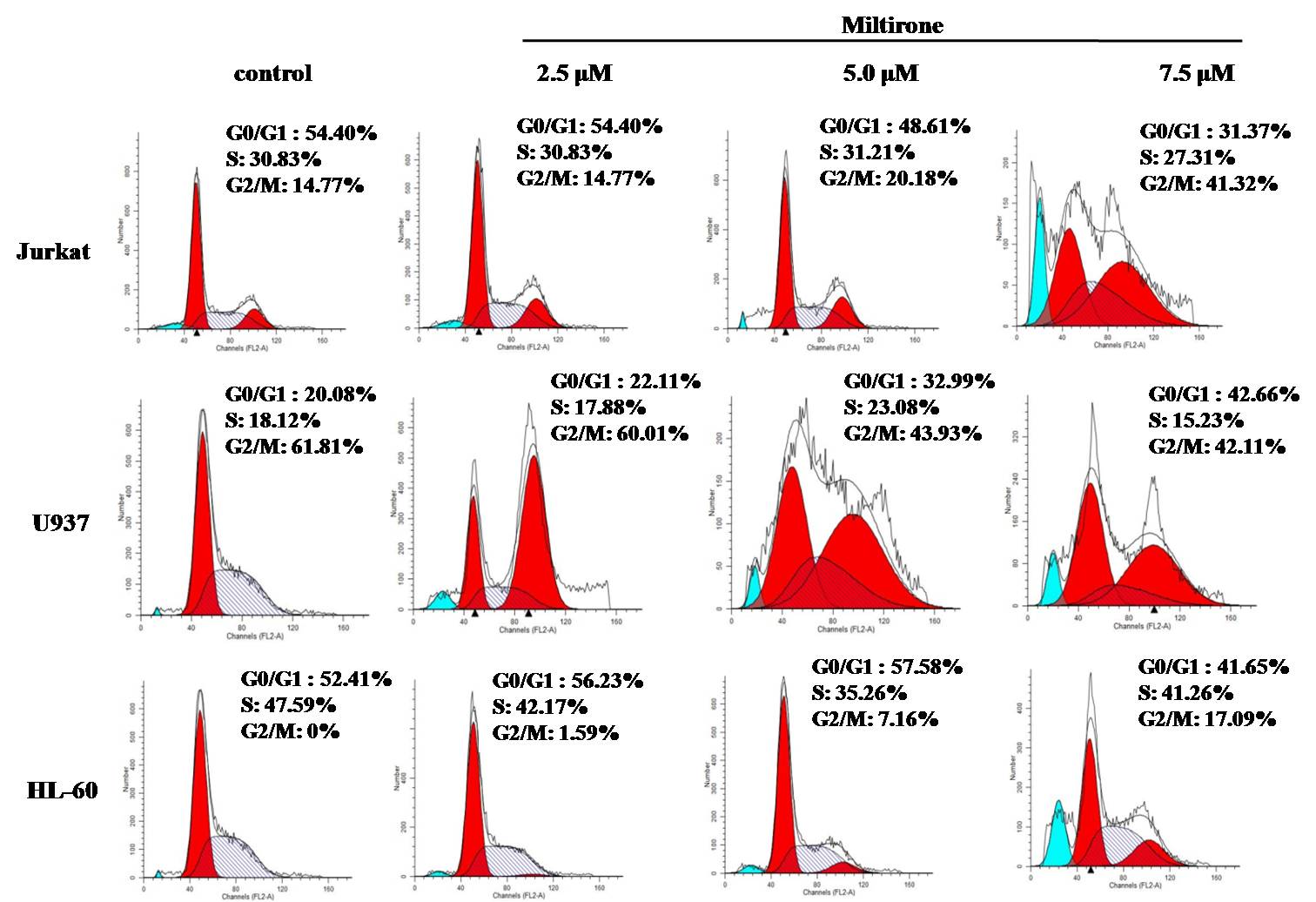


**Supplementary Fig. S3 Miltirone induces cell-cycle arrest in human leukemia cells.** Percentage cell-cycle distribution in cells following miltirone treatment for 12 h at indicated concentrations and FACS analysis was performed.


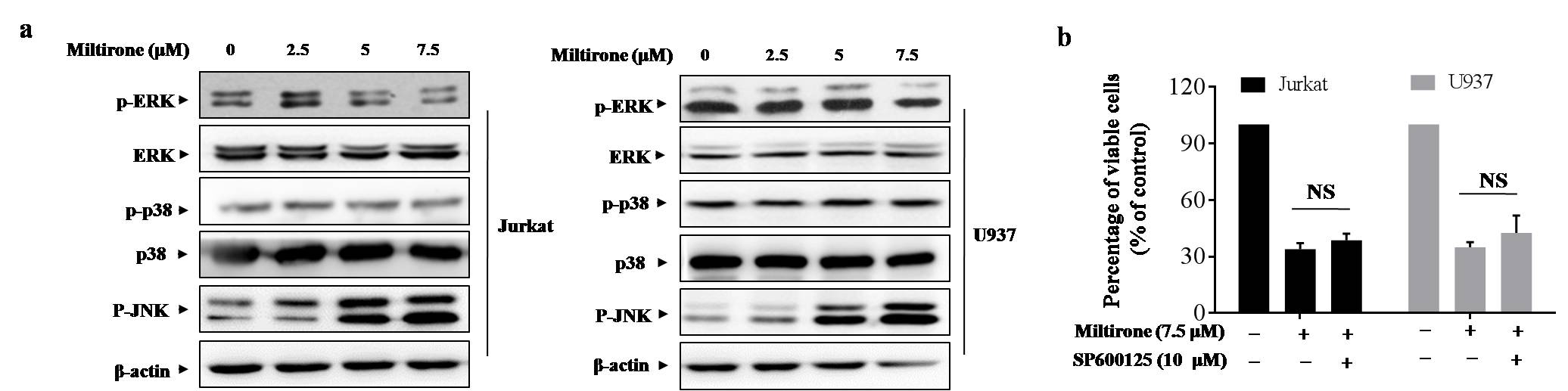


**Supplementary Fig. S4 JNK pathway is involved in the apoptosis induced by miltirone**. (a) p-ERK, ERK, p-p38, p38, p-JNK and β-actin protein expression were identified by western blot analysis after treatment of 12 h. (b) Effect of JNK inhibitor on miltirone -induced cell death. Cells were treated with 7.5 μM miltirone for 12 h in the presence or absence of 10 μM SP600125 and cell viability was determined by CCK8 assay. Data were presented as Mean ± SD. The differences were no significant (NS) at p > 0.05 vs. control.


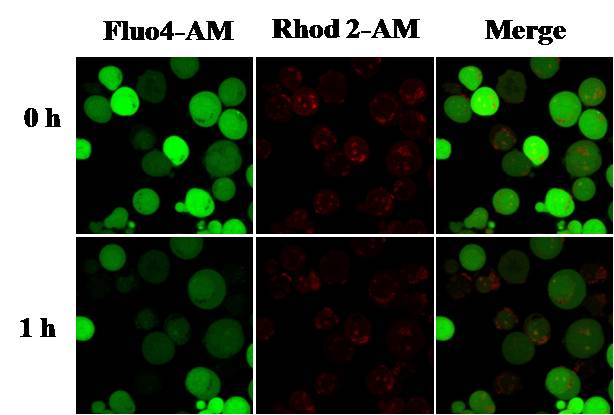


**Supplementary Fig. S5** **The calcium distribution imaging was measured by confocal microscopy in control group.** Jurkat cells were loaded with cell-permeant calcium indicators, 2 μM of Fluo 4-AM (DOJINDO Laboratories) and 2 μM of Rhod 2-AM (DOJINDO Laboratories), in HBSS for 30 min at 37°C. The calcium distribution imaging was measured by confocal microscopy.

**Reference**

1. Liu, H. *et al.* Oligosaccharide G19 inhibits U-87 MG human glioma cells growth in vitro and in vivo by targeting epidermal growth factor (EGF) and activating p53/p21 signaling. *Glycobiology* **24**, 748-765, doi:10.1093/glycob/cwu038 (2014).
